# Severe Methylmalonic Acidemia Precipitated by Dietary B12 Deficiency in Vegan Toddler

**DOI:** 10.3390/pediatric18040108

**Published:** 2026-08-06

**Authors:** April Edwell, Susan Bessler, Shannon Burke

**Affiliations:** Department of Pediatrics, University of California, San Francisco, CA 94158, USA; susan.bessler@ucsf.edu (S.B.); shannon.burke@ucsf.edu (S.B.)

**Keywords:** B12 deficiency, methylmalonic acidemia, vegan diet

## Abstract

A previously healthy 19-month-old female presented after her family found her upon awakening to be sleepy, uninterested in eating, and lethargic. On arrival, blood gas showed profound metabolic acidosis with normal lactate that did not improve with normalization of glucose and sodium. Her acidosis continued to worsen, so a broad workup was initiated, considering ingestions, DKA, and inborn errors of metabolism. In collecting further history, the patient’s nutrition history included a combination of breastfeeding and a vegan diet followed by her family. Her B12 level returned undetectably low. She improved dramatically over the next several hours after receiving an injection of cyanocobalamin. Ultimately, she was confirmed to have methylmalonic acidemia secondary to severe Vitamin B12 deficiency.

## 1. Introduction

Vitamin B12, or cobalamin, is necessary for normal erythropoiesis and neurologic function. B12 is a critical cofactor for two enzymes in the body: methionine synthase and methylmalonyl-CoA mutase. Because methionine synthase is vital for normal DNA synthesis in proliferating cells and one-carbon metabolism, its failure has a pronounced effect, especially for hematopoiesis, leading to dysplastic marrow that leads to megaloblastic anemia [1,2]. Without normal methionine production, myelin synthesis and maintenance can be defective, as can normal neurotransmitter synthesis, resulting clinically in a range of neurologic symptoms including impaired cognition and development, neuropathy, myelopathy, and paresthesia [3,4]. Importantly, B12 deficiency also leads to pathologic accumulation of homocysteine, which is associated with thrombosis, and of methylmalonic acid, which can cause profoundly elevated AG acidosis.

It is critical for pediatric providers to be able to prevent, identify and treat B12 deficiency, as unchecked it can lead to bone marrow failure and irreversible neurologic impairment, but with early treatment it is largely reversible [1,5,6]. Because B12 is synthesized by microorganisms and is naturally present in meaningful amounts primarily in animal products, individuals following plant-based diets require reliable intake from fortified foods or supplements. Here, we present the case of a breastfed 19-month-old following a vegan diet who presented with lethargy, hypotonia and profound acidosis from methylmalonic acidemia secondary to severe B12 deficiency.

## 2. Patient Presentation

A previously healthy 19-month-old female was brought in for evaluation by her family after she awoke one morning seemingly tired and uninterested in moving or eating. She had been in her usual state of health the prior evening. On arrival to the Emergency Department, she was noted to be lethargic and breathing rapidly. Initial labs revealed profound acidosis with a pH of 7.1 and a large base deficit with a normal lactate (Table 1). She was also hypoglycemic and hyponatremic. She was given a dextrose bolus and intravenous fluids with notable improvement in electrolytes, but her mental status did not improve, so she was transferred to the Pediatric Intensive Care Unit (ICU).

On arrival to the ICU, she remained lethargic and was noted to have a Kussmaul breathing pattern. Her extremities were cool, and she displayed diffuse hypotonia, though she was opening her eyes spontaneously and pushing away examiners. Initial capillary gas showed worsening acidosis (Table 2). After more isotonic fluid resuscitation, her pH improved slightly, but her mental status was unchanged. Anion gap (AG) was elevated at 29.

## 3. Diagnostic Assessment

We began a broad workup for severe metabolic acidosis. Given the degree of acidosis and her Kussmaul breathing, we considered Diabetic Ketoacidosis (DKA), despite her young age and presenting hypoglycemia. Her serum ketones returned elevated, prompting consideration of starvation ketosis, but there was no history of decreased intake or growth challenges, and she did not improve over several hours despite glucose correction and fluid resuscitation. Her urine toxicology screen, acetaminophen, and salicylate levels were negative, but we still had a high suspicion for ingestion, particularly ethylene glycol, given the elevated AG. However, we consulted with Poison Control and determined that because her osmolar gap was low and she had normal renal function and robust urine output, ethylene glycol toxicity was unlikely.

We also considered atypical presentations of inborn errors of metabolism. In our initial blood draw, we sent plasma amino acids, acylcarnitine profile, carnitine, ammonia, and urine organic acids. The amino and organic acids had not resulted the night of presentation, though the ammonia returned within an hour as slightly elevated. However, the absence of significant lactic acidosis and the rapid resolution of hypoglycemia with a single dextrose bolus, without recurrence on maintenance fluids, made an inborn error of metabolism unlikely.

The family denied recent new exposures or opportunities for occult ingestion and reported no recent dietary changes. Her diet consisted of breastfeeding and a vegan diet of grains, fruits, vegetables, and quinoa, without fortified foods or plant-based milks. She was not receiving vitamin or mineral supplementation. On review of her initial complete blood count, she was noted to be anemic with an elevated mean corpuscular volume despite having iron deficiency and an elevated number of segmented neutrophils on the automated differential, concerning for megaloblastic anemia. This prompted us to send a B12 level with our initial labs—it returned within a few hours, critically low (Table 3). This pushed us to reinterpret her labs through the lens of profound B12 deficiency, suggesting her profound acidosis was a result of methylmalonic acidemia (MMA), which ultimately returned several days later severely elevated.

## 4. Treatment and Outcome

We promptly gave her a dose of 1000 mcg of cyanocobalamin. Within a few hours, her mental status dramatically improved, and her acidosis completely resolved. Later, her homocysteine level returned elevated, as did her serum methylmalonic acid, confirming a diagnosis of MMA. She began ongoing B12 supplementation during her hospitalization and by the time of discharge a few days later was at her mental and temperamental baseline, though she had some ongoing weakness and instability with ambulation, which will be followed and treated as an outpatient. Her family readily received resources about both naturally occurring and supplemental vegan sources of B12 (Table 4) as well as recommended intake (Table 5). She was also started on an iron-containing multivitamin and vitamin D supplementation with plans to have her PCP follow her nutritional and hematologic labs. Her parent’s written consent was obtained for publication of this case.

## 5. Discussion

B12 deficiency is an important pediatric public health problem, with a prevalence ranging from 5% to up to 80%, with the higher rates noted in resource-constrained settings [2,3,9]. Causes of B12 deficiency in infants and children include insufficient dietary intake, poor bioavailability, and impaired absorption. While in adults this is classically pernicious anemia from lack of intrinsic factor, in pediatric populations it includes patients with altered GI anatomy and function (gastrectomy, gastritis, or gastric bypass, pancreatic insufficiency, ileal resection/short gut, and Crohn’s and celiac disease), those with interference from certain medications (gastric acid blockers, metformin, or nitrous oxide), some tapeworm infections and bacterial overgrowth [1,3,5,10]. Infants are at increased risk for dietary deficiency as they are reliant on parental B12 supply in utero. Furthermore, the B12 content of breast milk is dependent on the B12 status of the lactating parent and may be insufficient to meet infant requirements when the parent is also B12 deficient, placing exclusively breastfed infants at increased risk for deficiency [6].

Globally, dietary B12 deficiency correlates with resource-constrained populations who may have a low consumption of animal-source foods [7]. However, as plant-based diets gain popularity, it is critically important to consider B12 deficiency in well-resourced populations as well. In the US over the past 2 decades, there has been an estimated 20% increase in people choosing plant-based diets, defined as ≥50% total protein from plants [11]. In the case described here, the caregivers opted to include their child in the vegan diet they had chosen for themselves. As caregiver and child dietary patterns are often similar, the rise in plant-based diet choices in adults has important ramifications for pediatric providers [12].

In this case, the patient had weakness and hypotonia, consistent with the disruption in methionine synthesis, as well as megaloblastic anemia despite concurrent iron deficiency (which usually presents with microcytic anemia). Her B12 deficiency was severe enough that she had profound elevation of her methylmalonic acid toward the highest end of what is commonly described in the literature [1].

This case underscores the importance of including B12 deficiency-related MMA in the differential of acidosis, because timely supplementation can prevent progression to irreversible neurologic damage. This patient’s initial presentation with acute encephalopathy and low pH led to a prompt evaluation of high AG metabolic acidosis, a common clinical scenario for pediatric intensivists. However, most mnemonics and workup algorithms for AG metabolic acidosis do not include B12 deficiency precipitated MMA explicitly [13]. Most place MMA (if included at all) in the bucket with inborn errors of metabolism. In this case, we quickly considered sepsis/hypoperfusion, starvation ketosis, DKA, inborn errors of metabolism, and ingestion. However, the history, response to therapies, and lab testing were not a good fit for any of them, and it was not until noting the megaloblastic anemia paired with the diet history that B12 deficiency entered the differential as a uniting explanatory framework. This highlights the importance of avoiding premature closure, as well as the critical need to expand and revise our most common clinical reasoning paradigms, especially given the increasing incidence of plant-based diets.

Furthermore, this case highlights the importance of prevention. Primary care providers should routinely assess dietary patterns, use of fortified foods, and micronutrient supplementation. This patient had normal growth parameters (height, weight, and BMI), so a nutritional deficiency could have been missed without focused screening and history-taking beyond the physical exam. Infants and children following vegan dietary patterns should have careful monitoring of B12 intake and may require laboratory evaluation including serum B12, complete blood count, and confirmatory testing with methylmalonic acid or homocysteine when deficiency is suspected. Because vitamin B12 is not reliably obtained from unfortified plant foods, children following vegan dietary patterns require regular intake of B12-fortified foods and/or vitamin B12 supplementation [8].

In summary, we report a case of profound B12 deficiency in a vegan breastfed toddler leading to severe methylmalonic acidemia, lethargy, and hypotonia. Testing demonstrated an undetectable serum B12 level, as well as elevated homocysteine and methylmalonic acid levels, and her hemogram showed megaloblastic anemia. Her acidosis and metal status responded rapidly to B12 supplementation. As veganism and other plant-based dietary patterns have become increasingly common among children and families, pediatric providers must be equipped to recognize both the risk factors and signs and symptoms of B12 deficiency. Routine dietary assessment, nutrition counseling, targeted laboratory screening, and swift treatment may prevent irreversible neurologic complications.

## Figures and Tables

**Table 1:** Laboratory results in the emergency department.

Laboratory Test	Value	Reference Range
Sodium, mEq/L	127	136–145
Potassium, mEq/L	3.4	3.4–4.5
Chloride, mEq/L	94	98–110
Bicarbonate, mEq/L	4	21–32
BUN, mg/dL	28	7–18
Creatinine, mg/dL	0.24	0.55–1.02
Glucose, point-of-care, mg/dL	28	74–106
Glucose, venous (after dextrose), mg/dL	149	74–106
Calcium, mg/dL	8.9	8.5–10.1
Anion gap	29	10–20
pH, venous	7.10	7.31–7.40
pCO_2_, venous, mmHg	21	30–50
Base Excess, venous	−21	−2.0–2.0
Lactate, mmol/L	1.3	0.9–1.7

**Table 2:** Laboratory Results on arrival to PICU.

Laboratory Test	Value	Reference Range
Sodium, mmol/L	136	135–145
Potassium, mmol/L	3.1	3.5–5.3
Chloride, mmol/L	103	98–110
Bicarbonate, mmol/L	5	20–28
BUN, mg/dL	17	7–18
Creatinine, mg/dL	0.22	0.10–0.40
Glucose, mg/dL	176	70–140
Calcium, mg/dL	8.6	8.0–11.0
Anion gap	28	7–18
Osmolar gap	3	−14–10
pH, venous	7.02	7.31–7.47
pCO_2_, venous, mmHg	10	30–50
Base excess, venous	−28	−2.0–3.0
Lactate, mmol/L	1.7	0.36–1.25
WBC, x10E9/L	16.6	5.0–15.0
Hemoglobin, g/dL	10.5	11.0–15.8
Hematocrit, %	30.7	33.0–40.0
Platelet count, x10E9/L	334	150–400
MCV, fL	97	73–88

**Table 3:** Initial nutritional and other notable labs.

Laboratory Test	Value	Reference Range
B12, pg/mL	<150	211–946 *
Iron, serum, mg/dL	25	37–145
Folate, ng/mL	39.2	5.4–20.0
Beta-hydroxybutyrate (serum ketones), mmol/L	6.48	0.02–0.27
Ammonia, mmol/L	63	29–59
Vitamin D, 25-Hydroxy, ng/mL	12	30–96
Homocysteine, µmol/L	32.2	3.3–7.2
Methylmalonic Acid, Serum, nmol/L	180,400	55–347
C3, Propionylcarnitine µmol/L	4.86	0.08–1.77
Carnitine, Free µmol/L	3.8	18.0–58
Acylcarnitine/free carnitine ratio	4.9	0.1–0.4

* Per Elecsys vitamin B12 assay, which uses a competitive electrochemiluminescence immunoassay (ECLIA) principle where endogenous vitamin B12 in the sample competes with added biotin-labeled vitamin B12 for limited binding sites on a ruthenium-labeled intrinsic factor complex.

**Table 4:** Plant-based sources of B12 [7,8].

Category	Examples	Notes
Naturally occurring foods	Dried seaweed (Aonori, maesangi, nori)	Limited amounts; varies by growing conditions
	Mushrooms (Shiitake, golden chanterelle, and black trumpet)	Limited amounts; varies by growing conditions
	Fermented tempeh	If made with B12-producing bacteria
	Fermented soy yogurt	If made with B12-producing bacteria
Fortified foods	Fortified plant-based milk	Check label for amount, varies by product
	Fortified breakfast cereals	Check label for amount, varies by product
	Fortified nutritional yeast	Check label for amount, varies by product

**Table 5:** Recommended intake of B12 by age, adapted from Larvie et al. [3].

Age Group	Daily Requirement
0–6 months	0.4 µg/day (AI)
7–12 months	0.5 µg/day (AI)
1–3 years	0.9 µg/day (RDA)
4–8 years	1.2 µg/day (RDA)
9–13 years	1.8 µg/day (RDA)

AI = Adequate intake; RDA = Recommended dietary allowance.

## Data Availability

No new data were created or analyzed in this study. Data sharing is not applicable to this article.

## Abbreviations

The following abbreviations are used in this manuscript:

DKA	Diabetic ketoacidosis
MMA	Methylmalonic acidemia
AG	Anion gap
ICU	Intensive care unit
